# Reconstruction of Oncologic Sternectomy Defects: Lessons Learned from 60 Cases at a Single Institution

**DOI:** 10.1097/GOX.0000000000002351

**Published:** 2019-07-26

**Authors:** Joseph Banuelos, Amjed Abu-Ghname, Uldis Bite, Steven L. Moran, Karim Bakri, Shanda H. Blackmon, Robert Shen, Mark S. Allen, Peter C. Pairolero, Philip G. Arnold, Basel Sharaf

**Affiliations:** From the * Division of Plastic Surgery, Department of Surgery, Mayo Clinic, Rochester, Minn.; † Division of Thoracic Surgery, Department of Surgery, Mayo Clinic, Rochester Minn.

## Abstract

Oncologic sternectomy results in complex defects where preoperative planning is paramount to achieve best reconstructive outcomes. Although pectoralis major muscle flap (PMF) is the workhorse for sternal soft tissue coverage, additional flaps can be required. Our purpose is to evaluate defects in which other flaps beside PMF were required to achieve optimal reconstruction.

**Methods:**

A retrospective review of consecutive patients at our institution who underwent reconstruction after sternal tumor resection was performed. Demographics, surgical characteristics, and outcomes were evaluated. Further analysis was performed to identify defect characteristics where additional flaps to PMF were needed to complete reconstruction.

**Results:**

In 11 years, 60 consecutive patients were identified. Mean age was 58 (28–81) years old, with a mean follow-up of 40.6 (12–64) months. The majority were primary sternal tumors (67%) and the mean defect size was 148 cm^2^ (±81). Fourteen (23%) patients presented with postoperative complications, and the 30-day mortality rate was 1.6%. In 19 (32%) cases, additional flaps were required; the most common being the rectus abdominis muscle flaps. Larger thoracic defects (*P* = 0.011) and resections involving the inferior sternum (*P* = 0.021) or the skin (*P* = 0.011) were more likely to require additional flaps.

**Conclusions:**

Reconstruction of oncologic sternal defects requires a multidisciplinary team approach. Larger thoracic defects, particularly those that involve the skin and the inferior sternum, are more likely to require additional flaps for optimal reconstruction.

## INTRODUCTION

Tumors of the sternum are rare and can develop from primary bone pathology or through metastatic spread.^1,2^ Surgical management requires partial or total sternectomy; these defects create unique reconstructive challenges for the plastic surgeon as one must re-establish stability of the anterior chest wall.^2–4^ Wound coverage with local or free flaps focus on protecting underlying thoracic structures, in addition to supporting and stabilizing chest wall movement while maintaining pulmonary dynamics.^3,5–9^ Despite advances in reconstructive surgical techniques, imaging and surgical planning, sternal reconstruction continues to be challenging and lacks consensus on best practice.^10^ Although chest wall instability following sternotomy nonunion in the setting of infection remains an unsolved problem, postoncologic sternal defects pose an even greater challenge, especially when reconstructing large composite anterior chest wall defects. In addition, neoadjuvant chemotherapy and radiation to the tumor bed increase the risk of delayed wound healing.^11,12^

Traditionally, the pectoralis major flap (PM) has been the workhorse flap for reconstruction of postcardiac sternotomy wounds.^13–15^ Other reconstructive options include the rectus abdominis muscle (RAM) flap, omentum flap, or latissimus dorsi flap.^16–20^ Although these flaps continue to be valuable in postoncologic sternal defect reconstruction, optimizing flap selection and chest wall stability for anticipated sternal defects are still needed. Aside from a few case series, there are limited large studies of sternal reconstruction outcomes.^4,21^ In this study, we report our experience with reconstruction of sternectomy defects in 60 patients focusing on outcome and complications. Our purpose is to evaluate defects in which other flaps beside PMF were required to achieve optimal reconstruction.

## METHODS

A retrospective electronic chart review was performed to identify all consecutive patients who presented with either a primary or a metastatic sternal tumor at our institution from January 2000 to January 2017. Only adult patients, >18 years of age, who underwent sternal tumor resection and reconstruction were included. Patients were excluded if they were younger than 18 years of age at the time of surgery or did not consent to use of their medical records for research purposes. This study was approved by our Institutional Review Board.

Patient’s demographics including age, gender, body mass index, smoking status, previous radiotherapy, and medical comorbidities were collected. Tumor classification was based on final pathology report and primary or metastatic etiology. Surgical variables such as thoracic defect size, extent and location of defect, type of prosthetic material used for thoracic reconstruction, presence of skin defect and its size, internal mammary artery (IMA) status following tumor resection, and the utilized reconstructive approach were also reviewed. The IMA status was divided into 3 categories: Category (A) unilateral IMA present, (B) bilateral IMAs present, and (C) both IMAs absent.

Postoperative complications, including wound dehiscence, partial or complete flap necrosis, seroma, hematoma and surgical site infection (SSI) were collected. The Centers for Disease Control and Prevention criteria for SSI were used.^22^ Skin flap necrosis was defined as full-thickness skin necrosis that required debridement or wound care. Seroma and hematoma were defined as those that were treated by aspiration or evacuation in the operating room. Follow-up time, reoperation rate, 30-day and 1-year mortality rates were also evaluated.

## STATISTICAL ANALYSIS

Continuous data were presented as medians with interquartile ranges for the 25–75th percentiles, and comparisons were performed with the Mann–Whitney–Wilcoxon test. Categorical data were presented as percentages and analyzed using the chi-square test, and for small samples, Fisher’s exact test was used. Finally, data were analyzed to compare defects in which PMF was used primarily with those that required additional flaps to complete the reconstruction. A *P* value of <0.05 was considered significant. Statistical analysis was performed using JMP Pro 13 software (JMP, Pro 13, SAS Institute Inc, Cary, N.C., 1989–2019).

## RESULTS

Patient’s characteristics are summarized in Table 1. A total of 60 consecutive patients were identified in the study period. These included 30 males and 30 females, with a median age of 58 (44–70) years old. The mean patient’s body mass index was 28 (±5) kg/m^2^. There were 5 (8.3%) active smokers and 24 (40%) patients with history of smoking. Sixteen (26.7%) patients had at least one comorbidity and 19 (32%) underwent preoperative radiotherapy.

**Table 1.**
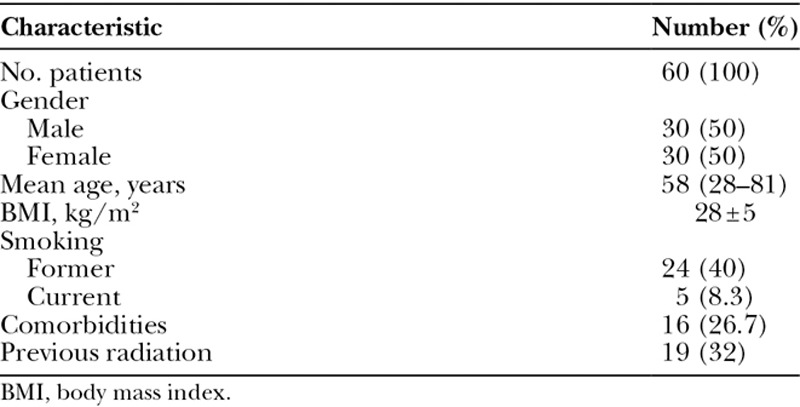
Patient Characteristics

Tumor characteristics are summarized in Table 2. The majority of sternal tumors (n = 40, 67%) were primary sternal tumors, whereas the remaining 20 (33%) were metastatic tumors. Sarcomas encompassed the most common type of primary tumor (67.5%) followed by desmoid tumors (12.5%). In addition, 4 (10%) advanced stage skin malignancies with sternal extension were included, and 4 (10%) were grouped as others, including 2 plasmacytomas, a neuroblastic tumor, and a calcifying fibrous tumor. Twenty (33%) patients presented with metastatic disease involving the sternum. Of the metastatic tumors, there were 8 (40%) breast cancer metastases, 5 (25%) papillary thyroid cancer, 4 (20%) renal tumors, and individual cases of prostate, hepatocellular carcinoma, and inflammatory myofibroblastic tumor.

**Table 2.**
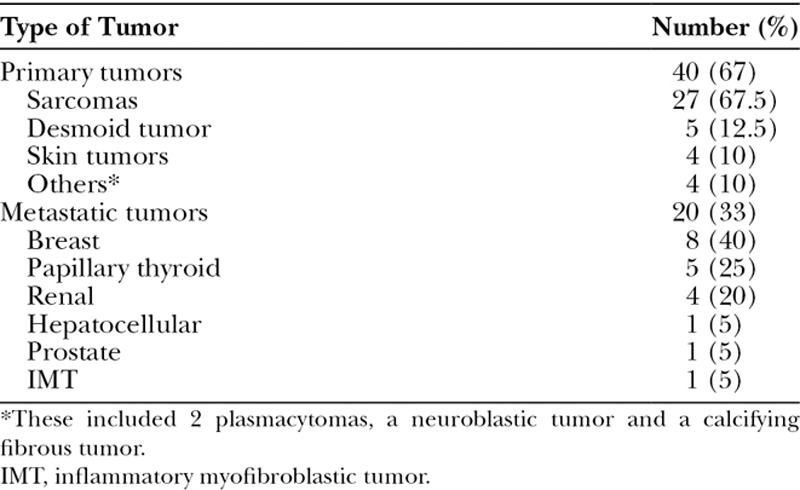
Sternal Tumor Characteristics

### Surgical Characteristics

**Fig. 1.**
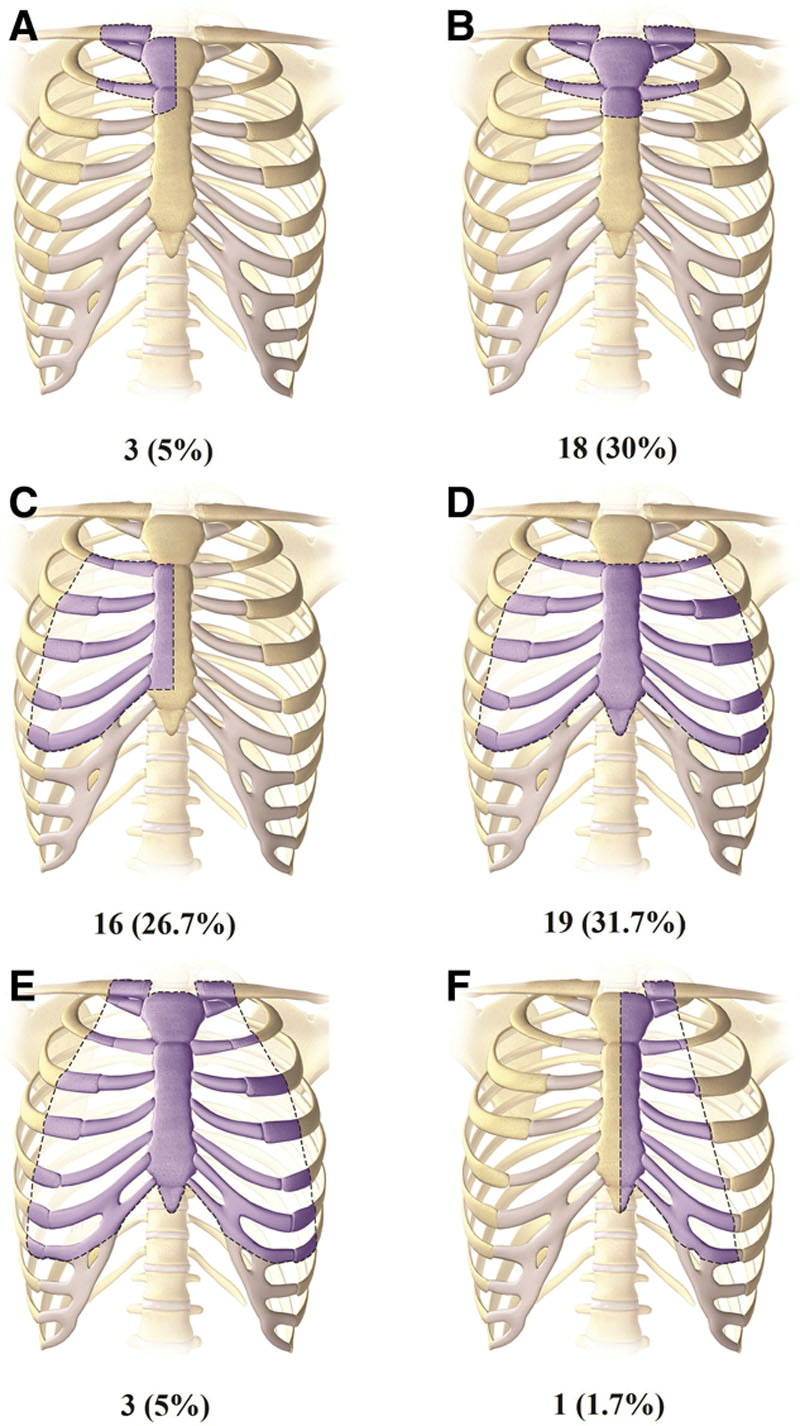
Thoracic defects of the patients. A, Resection involving half of the manubrium and adjacent sternum, clavicle heads, ribs, and cartilages. B, Resection involving all the manubrium and adjacent sternum with bilateral clavicle heads, ribs, and cartilage. C, Resection of half of the sternal body with adjacent unilateral ribs and cartilages. D, Resection of most of the sternal body with bilateral adjacent ribs and cartilages. E, Total sternectomy. F, Total hemisternectomy.

The extent of tumor resections varied and was grouped according to the size and location of the thoracic defect. The mean defect size was 148 cm^2^ (±81). Therapeutic (R0) resection was achieved in 55 (91%) of cases. In 5 (9%) patients, palliative resection was achieved and these included sternal tumors metastases from breast cancer, papillary thyroid cancer, basal cell carcinoma, osteosarcoma, and desmoid tumor. Table 3 summarizes patient’s surgical characteristics. In 52 (86%) patients, a prosthetic material was used for thoracic reconstruction. The most common prosthetic material used was Polytetrafluoroethylene (Gore-Tex, Flagstaff, Ariz.), in 46 (76.7%) of patients. After tumor resection, 17 (28%) patients had one IMA available, 22 (37%) had both IMAs available, and 21 (35%) had neither IMA available to aid in reconstruction. In 23 (38%) patients, skin was included with the tumor resection and the mean skin defect size was 85 cm^2^ (±112). Figure 1 summarizes the thoracic defects in our series and their relative frequencies.

**Table 3.**
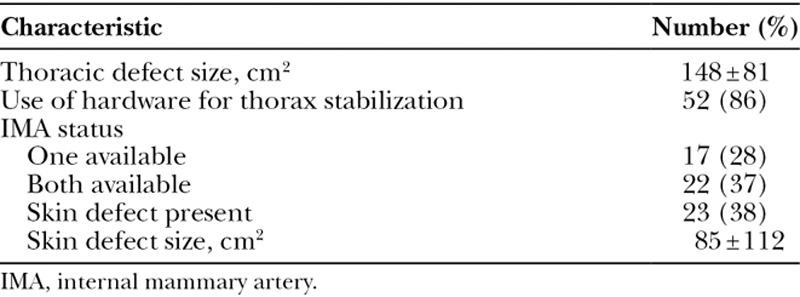
Surgical Characteristics

### Reconstructive Approach

**Fig. 2.**
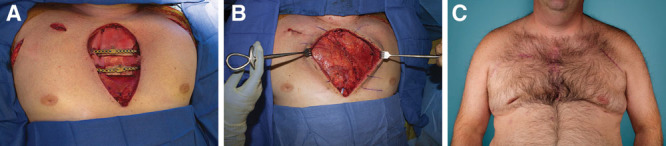
Patient with PMF reconstruction. A, A 49-year-old man status postmanubrial and upper sternal resection for chondrosarcoma, rigid fixation, and bone grafts. B, Bilateral PMF reconstruction for coverage of hardware. C, Two-year follow-up of the patient.

**Fig. 3.**
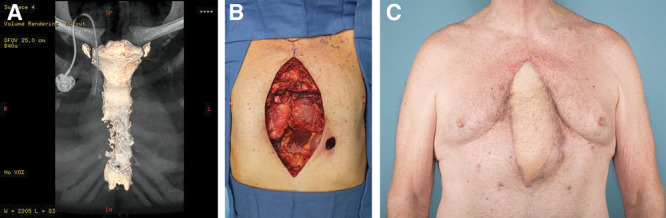
Patient with ALT flap reconstruction. A, Computerized tomography scan with 3D reconstruction showing chondrosarcoma in the lower sternum of a male patient. B, Intraoperative photograph showing a defect after subtotal sternectomy. C, Patient at 1-year follow-up after free ALT flap reconstruction.

Table 4 summarizes the reconstructive approaches used. The pectoralis major muscle flap (PMF) was used alone to reconstruct 41 (68%) of defects, with 8 (13%) being unilateral and 33 (55%) being bilateral. Figure 2 shows a patient with bilateral PMF reconstruction. Conversely, in 19 (32%) patients, additional flaps were used for reconstruction. In these patients, flaps included: latissimus dorsi flap in 5 cases, thoracoabdominal flap in 4 cases, RAM flap in 5 cases [3 transverse RAM (TRAM), 2 vertical RAM (VRAM)], omental flap in 2 cases, and free anterolateral thigh (ALT) flap in 2 cases. Finally, 1 patient was treated with reverse abdominoplasty alone. Figure 3 shows a case with free ALT reconstruction.

**Table 4.**
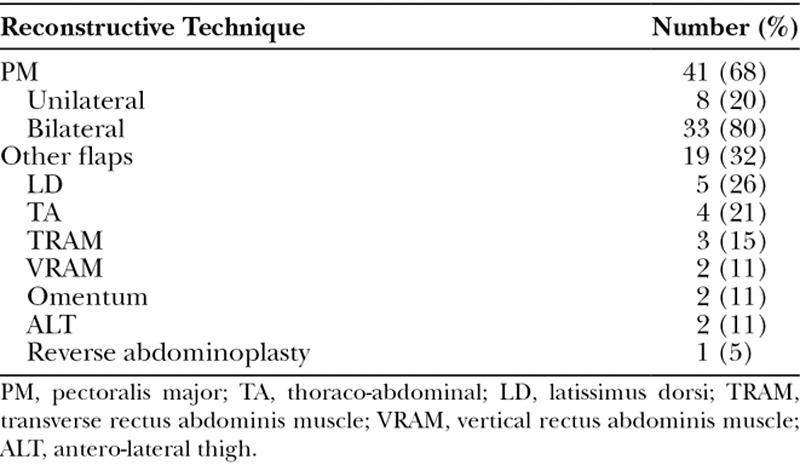
Reconstructive Techniques

### Surgical Outcomes

Patients were followed for a median of 40.6 (11.9–64.4) months. In this period, a total of 14 (23.3%) patients presented with surgical site complications which included: eleven (18%) SSIs, 1 (1.6%) primary wound breakdown, 1 (1.6%) flap venous congestion in a TRAM flap, and 1 (1.6%) partial flap necrosis in another TRAM flap. Patient’s presentation with SSI ranged from 9 to 276 days postoperatively (4 patients developed infection within the first month and 5 presented after 1 month of surgery). Eleven (18%) patients were taken back to the operating room for management of surgical site occurrences. These included 9 patients who presented with SSI, 1 patient with wound dehiscence, and 1 patient with partial flap necrosis. The remaining patients’ complications were managed nonoperatively. There were no cases that presented with hematomas or seromas. Table 5 summarizes surgical outcomes. One (1.6%) patient died within the first month of surgery due to pulmonary complications that resulted in respiratory failure. After 1 year of follow-up, 5 additional patients died for a total 1-year mortality rate of 10%. Progression of the disease was the primary cause of death in those patients, which included 2 patients with breast cancer, 1 with renal cell carcinoma, 1 with osteosarcoma, and 1 with basosquamous cell cancer.

**Table 5.**
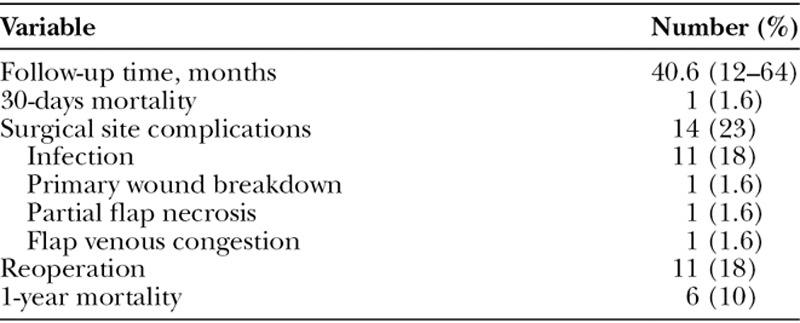
Surgical Outcomes

### Analysis of Reconstructive Approach

In the group of patients who required other types of tissue transfer, larger thoracic defects (*P* = 0.011), resections that involved the lower half of the sternum (*P* = 0.021), and composite resections that included skin (*P* = 0.011) were more common. No significant differences were present between the 2 groups in demographic characteristics, tumor types, or complications. Although surgical complications that required reoperations were more common when flaps other than PMF were utilized, this was not statistically significant. Table 6 lists cases in which the PMF was used alone and those which required other flaps to achieve reconstruction.

**Table 6.**
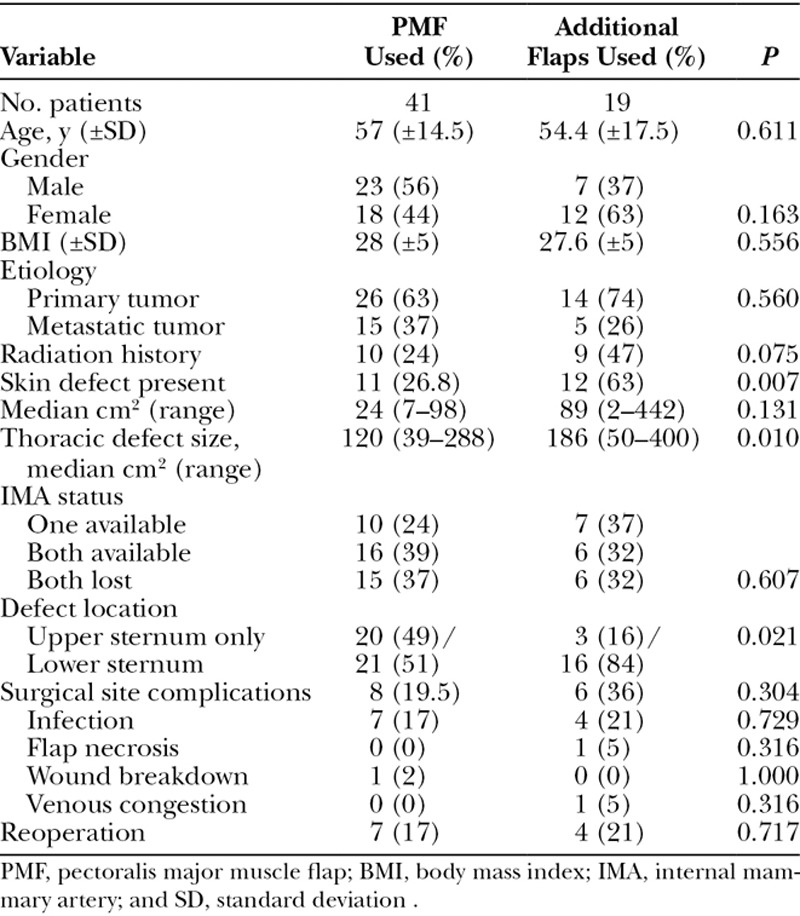
Analysis of Reconstruction Outcomes

## DISCUSSION

This retrospective review of our institution’s experience with oncologic sternal reconstruction following partial or total sternectomy highlights some of the reconstructive nuances in this rare patient population. Primary sternal tumors made up 67% of tumors resected and 77.5% of these primary tumors were sarcomas. The pectoralis major flap alone was the reconstructive modality utilized in 68% of defects while additional flaps were necessary in the remaining 32% of cases. Larger thoracic defects, composite resections that included skin and resections of the lower half of the sternum were significantly associated with the use of other flaps besides the PM to achieve successful reconstruction. The follow-up time ranged from 1 to 140 months with a median of 40.6 months (3.3 years) and presented a total complications rate of 23.3%. The most common complication was SSI with a rate of 18%. Four infections presented within the first 30 days and 5 presented after the first month of the surgery, ranging from 9 to 276 days. Reoperation rate and 30 days mortality were 18% and 1.6%, respectively. No significant differences in complications rate were found between the 2 groups.

Our study adds to the published literature with a series of 60 patients and longer evaluation times than previously published. Our study shows comparable complications and mortality rates with those previously reported.^4,23^ Butterworth et al^4^ reported a 38% complications rate in 49 patients who underwent reconstruction for oncological sternectomy, during a mean follow-up of 18 months. More recently, Bongiolatti et al^23^ reported outcomes on 36 sternectomy defects with a median follow-up of 24 months that showed a complication rate of 19%. Additionally, in-hospital and 30-day mortality rate reported in previous studies ranged from 0% to 9.5%,^3,4,21,23,24^ which was comparable to our 30-day mortality rate of 1.6%.

**Fig. 4.**
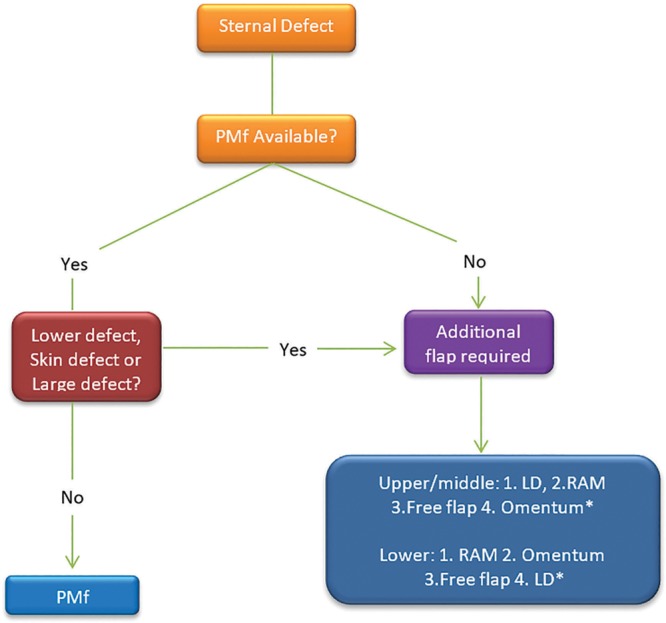
Algorithm for management of sternal defects. *Individual flap options may vary depending on defect and patient characteristics.

Soft tissue coverage of thoracic defects using muscle or myocutaneous flaps is an essential step that must be carefully considered and planned to achieve best outcomes.^16,25–28^ To date, published studies on oncologic sternal reconstruction have not focused primarily on comparing the indications and outcomes of the pectoralis major flap versus other flaps. Even though the PMF and RAM are considered among the top reconstructive options, the pectoralis major muscle is generally preferred if available.^13–15^ Although the PMF is generally the most commonly used flap for sternal reconstruction, it is not without limitations and in certain cases; the use of additional flaps becomes a necessity (Fig. 4). For patients with large defects, part of the sternocostal origins of the muscle may be resected with the tumor, which limits its reach of midline and inferior sternum defects. This concept was demonstrated in our study because additional flap options were likely to be used for larger defects and resections of the inferior sternum. This further demonstrates another important limitation for the pectoralis muscle flap for reaching inferior sternal or epigastric defects..^16–18,20,29–31^ In fact, these are the most common sites of wound dehiscence after surgical repair.^13,17,19,32,33^ Even though Hallock^34^ devised a technique of using a pectoralis major extended island flap with good results, the use of alternative flaps is generally considered in such inferior sternal defect cases.^17,18,20,30,31^

There have been numerous studies describing and advocating the use of alternative tissue transfer options for different sternotomy wounds.^13,17–20,30,31,33^ Davison et al^20^ in 2016 compared the results of 41 modified bilateral PMFs against 56 rectus abdominis muscle flaps in addressing infected lower third sternotomy defects following dehiscence. His study reported similar success, postoperative course, and morbidity and mortality rates between the two groups; both groups had a complication rate of 34%.^20^ In addition, it showed that even though the PMFs with rectus fascia extensions prevented superior dehiscence, this modification did not eliminate dehiscence of the lower third of the sternal wound.^20^ Our study compared 41 cases where no additional flaps besides PMF were required against 19 cases where other flaps were used. Our results show a complication rate of 22% in the PMF alone group versus 35.6% in the PMF with additional flaps group; however, this difference did not reach the significance level, making it consistent with the previously mentioned study.^20^ Moreover, specific complication rates also showed no significant correlation with any of the reconstructive techniques. Having similar complication and reoperation rates provide the reconstructive surgeon with a multitude of options when attempting to reconstruct a sternectomy defect following tumor resection.

As variable options are available for sternal reconstruction, careful multidisciplinary preoperative planning to achieve the best outcomes is paramount. Availability and state of the IM vessels has been previously shown that can affect the choice of the flap and the final outcome.^35–38^ Our study reported bilateral availability of IM vessels in almost one third of the defects (37%) and ipsilateral availability in 28%. This is comparable to previously reported IM status (26%) in one large series.^4^ Numerous studies have proved the crucial role of skeletal chest wall stabilization and reconstruction in determining postoperative morbidity and mortality, especially in wide anterior defects and in lateral chest wall defects larger than 5 cm.^5–9,23,39–42^ Extensive chest wall defects may affect respiratory function and chest wall stability.^5,8,9,39,43^ In our study, 52 (86%) of defects required chest wall stabilization with additional materials, the most common being polytetrafluoroethylene. Our results are comparable to those published by other US cancer centers such as Memorial Sloan Kettering Cancer Center, which reported an overall rate of 79.8% of prosthetic material use and median defect size of 80 cm.^2,5^ Additionally, MD Anderson Cancer Center, in 2013, reported the use of prosthetic material for thoracic stabilization in 82% of their cases; however, their most commonly used approach was rigid fixation using polymethylmethacrylate/polypropylene sandwich (37%).^4^ Despite the difference in the type of material used, our study demonstrated comparable complication rates.

To our knowledge, this is the largest series published on oncologic sternal reconstruction to date. However, it has several limitations. These include the relatively small sample size, which makes some comparisons underpowered. Its single-institution retrospective design has the potential for selection bias. Patient’s mortality resulted in limited follow-up time for some patients. In addition, there is a lack of follow-up data regarding musculoskeletal and pulmonary functional outcome. Despite these limitations, we provide a relatively larger sample size and longer follow-up time compared to available literature. Further multicenter prospective studies with larger sample size are necessary to provide evidence-based long-term results and comparisons.

## CONCLUSIONS

Reconstruction of oncologic sternal defects can be achieved successfully with acceptable complication rates when a multidisciplinary team approach is utilized. The reconstructive approach should be planned according to the anticipated defect characteristics. Larger thoracic defects, particularly those that involve the skin and the lower sternum, require additional flaps besides the pectoralis major muscle for optimal reconstruction.

## ACKNOWLEDGMENT

We thank Frank M. Corl, MS, for his help with illustrations.
